# Comparison of invasive fungal diseases between patients with acute myeloid leukemia receiving posaconazole prophylaxis and those not receiving prophylaxis: A single-center, observational, case-control study in South Korea

**DOI:** 10.1097/MD.0000000000025448

**Published:** 2021-05-21

**Authors:** Eunmi Yang, Eun-Ji Choi, Han-Seung Park, Sang-Oh Lee, Sang-Ho Choi, Yang Soo Kim, Jung-Hee Lee, Je-Hwan Lee, Kyoo-Hyung Lee, Sung-Han Kim

**Affiliations:** a Department of Infectious Diseases; b Hematology, Asan Medical Center, University of Ulsan College of Medicine; c Present affiliation: Department of Infectious Diseases, Seoul Medical Center, Seoul, Republic of Korea.

**Keywords:** Invasive fungal infections, posaconazole, acute myeloid leukemia, prophylaxis

## Abstract

Posaconazole prophylaxis is effective in decreasing the incidence of invasive fungal diseases (IFDs) in patients with acute myeloid leukemia (AML). However, the use of antifungal prophylaxis varies in real-life practice, and only a small number of studies have compared the incidence of IFDs between those receiving posaconazole prophylaxis and those without prophylaxis. We compared the clinical characteristics and outcomes of IFDs between patients with AML who received posaconazole prophylaxis and those without antifungal prophylaxis.

We reviewed the medical records of adult AML patients who underwent induction chemotherapy between June 2016 and October 2019 at Asan Medical Center (Seoul, South Korea), where posaconazole prophylaxis is not administered in patients with gastrointestinal symptoms that may hinder sufficient absorption of oral prophylactic agents, and in patients with abnormal liver functions considering the possible exacerbation of adverse events. Patients who received posaconazole prophylaxis for ≥7 days were included in the prophylaxis group. Clinical characteristics and outcomes including the incidence of IFDs were compared between the 2 groups.

Of the 247 patients with AML who underwent induction chemotherapy, 162 (66%) received posaconazole prophylaxis and 85 (34%) did not receive any prophylaxis. The incidence of proven/probable IFD was significantly higher in the no prophylaxis group than in the prophylaxis group (9.4% [8/85] vs 2.5% [4/162], *P* = .03). Of the 8 cases of IFDs in the no prophylaxis group, 7 were mold infections and 1 was invasive candidiasis. Of the 4 cases of IFDs in the prophylaxis group, 3 were mold infections and 1 was invasive candidiasis. Patients with posaconazole prophylaxis less frequently received therapeutic antifungal therapy (2.5% vs 9.4%, *P* = .03) and had a longer median, duration from chemotherapy to antifungal therapy compared with the no prophylaxis group (18 vs 11 days, *P* < .01). The rate of IFD-related mortality was similar between the 2 groups (0.6% vs 0%, *P* > .99).

Patients with AML who received posaconazole prophylaxis had a lower incidence of breakthrough IFDs compared with those who did not receive any prophylaxis. Invasive mold infection was the most common IFD regardless of antifungal prophylaxis.

## 1 Introduction

Invasive fungal diseases (IFDs) are important causes of mortality and morbidity in patients with hematologic malignancies.^[1]^ Due to prolonged bone marrow aplasia resulting from induction chemotherapy or hemopoietic stem cell transplantation (HSCT), patients with acute leukemia have particularly high risks for IFDs.^[2]^ As such, the incidence of IFDs has been reported to be as high as 24% to 36% among patients with leukemia who have received intensive induction chemotherapy or HSCT.^[3,4]^ Moreover, the rate of mortality from IFDs is high because the diagnosis of IFDs is often delayed, and antifungal prophylaxis is commonly used for the prevention of IFDs.^[2,5]^

Fluconazole has been used as the standard antifungal prophylactic agent for reducing morbidity and mortality among patients with hematologic malignancy.^[3,6,7]^ However, a randomized controlled trial demonstrated that posaconazole prophylaxis was more effective than fluconazole or itraconazole in preventing IFDs in patients with acute myeloid leukemia (AML)^[8]^; as such, current guidelines recommend posaconazole as the drug of choice for antifungal prophylaxis in patients with AML undergoing induction chemotherapy or HSCT.^[8–10]^ Accordingly, many studies compared posaconazole with other antifungal prophylaxis in terms of the incidence of IFD in patients with AML.

In real practice, however, the adoption of antifungal prophylaxis varies in real practice depending on the type of chemotherapy, expected duration of neutropenia, concern of adverse drug reactions, and health insurance coverage. Unfortunately, studies comparing the incidence of IFD between those receiving posaconazole prophylaxis and those without prophylaxis in real clinical practice are lacking. A few studies compared the incidence of IFD between those who received posaconazole prophylaxis and those without antifungal prophylaxis, but they either had a small number of study patients or had differences in the study period between the study group and the control group.^[6,11]^ At Asan Medical Center, posaconazole prophylaxis is not administered in patients with gastrointestinal symptoms that may hinder sufficient absorption of oral prophylactic agents, and also in patients with abnormal liver functions considering the possible exacerbation of adverse events associated with prophylaxis. As such, we had a unique opportunity to evaluate the incidence of breakthrough IFDs in patients given posaconazole prophylaxis compared with those without antifungal prophylaxis.

## 2 Patients and methods

### 2.1 Study population and design

We retrospectively reviewed the medical records of adult patients (age ≥18 years) with AML who received induction chemotherapy between June 2016 and October 2019 at Asan Medical Center, a tertiary hospital in Seoul, South Korea. Patients with AML who were undergoing remission-induction and reinduction therapy after relapse were also included. For patients who received repeated remission-induction chemotherapies, only the first treatment episode was analyzed.

At Asan Medical Center, posaconazole prophylaxis was administered at the discretion of attending hematologists based on the presence of gastrointestinal symptoms and abnormal liver functions considering possible malabsorption of oral prophylactic agents and exacerbation of adverse events. Prophylaxis was administered prior to chemotherapy or within 24 hours after the last cytotoxic chemotherapy and was continued until recovery from neutropenia or until the occurrence of IFDs. Patients in the prophylaxis group received a loading dose of 300 mg of posaconazole delayed-release tablets twice a day, followed by a maintenance dose of 300 mg once daily. Patients with a history of IFDs, prophylaxis with drugs other than posaconazole, or antifungal prophylaxis for <7 days were excluded. This study was approved by the Institutional Review Board of Asan Medical Center (IRB number 20191470).

### 2.2 Diagnostic workup of IFDs

Diagnostic workups included chest radiography and blood cultures at the onset of fever, and serum galactomannan assay and chest computed tomography (CT) for IFDs when patients had symptoms or signs suggestive of IFDs such as newly developed pneumonia and no resolution of neutropenic fever within 4 to 7 days of initial empirical antibacterial agent administration. Additional examinations (e.g., bronchoalveolar lavage, sinus or brain CT, abdominal CT or sonography, skin biopsy, video-assisted thoracic surgery [VATS] biopsy, endoscopic sinus surgery biopsy, and fundus examination) were performed as needed.

### 2.3 Definitions and endpoint

Neutropenia was defined as absolute neutrophil count <500 cells/mm^3^, and fever in neutropenic patients was defined as a single oral temperature of ≥38.3 °C or a sustained temperature of ≥38.0 °C for over 1 hour.^[12]^ IFDs were defined according to the definition of the European Organization for Research and Treatment of Cancer and the Mycoses Study Group Education and Research Consortium (EORTC/MSGERC).^[13]^ AML was classified according to the 2016 World Health Organization (WHO) classification.^[14]^ AML classification and chemotherapy regimens were individually discussed among the hematologists.

The primary endpoint was the incidence of IFDs. Breakthrough IFDs were defined as proven or probable IFDs developing after ≥7 days of antifungal prophylaxis. Secondary endpoints included the use of empirical antifungal agents, use of therapeutic antifungal agents, duration from initial chemotherapy to empirical or therapeutic antifungal agent use, and IFD-related mortality. Empirical antifungal agents were administered in patients with persistent neutropenic fever after 4 to 7 days of administration of a broad-spectrum antibacterial agent or those with suspected IFDs.

### 2.4 Statistical analysis

The posaconazole prophylaxis group and no antifungal prophylaxis group were compared in this study. Categorical variables were compared using the Chi-Squared test or Fisher exact test, and continuous variables were analyzed using the Student *t* test and Mann–Whitney *U* test, as appropriate. All statistical tests were 2-tailed, and *P* values <.05 were considered statistically significant. IBM SPSS Statistics for Windows, version 21.0 (IBM Corp., Armonk, NY) was used for statistical analysis.

## 3 Results

### 3.1 Patient characteristics

From June 2016 to October 2019, a total of 247 patients were included in the study. Of these patients, 162 (66%) received posaconazole prophylaxis and 85 (34%) did not receive any antifungal prophylaxis. Baseline characteristics of the patients are summarized in Table 1. There were no significant differences in age, gender, underlying diseases, and duration of neutropenia (Table 1).

**Table 1:** Clinical characteristics of patients with posaconazole prophylaxis and those without prophylaxis.

Characteristic	Posaconazole prophylaxis (n = 162)	No prophylaxis (n = 85)	*P* value
Age, median yr (IQR)	54 (43–61)	53 (43–61)	>.99
Male gender	82 (50.6)	51 (60.0)	.16
Underlying disease			
Acute myeloid leukemia	158 (97.5)	82 (96.5)	.70
Therapy-related myeloid neoplasms	3 (1.9)	1 (1.2)	>.99
Others^∗^	1 (0.6)	2 (2.4)	.27
Phase of chemotherapy			
Induction/re-induction	137 (84.6)	62 (72.9)	.028
Salvage induction	25 (15.4)	23 (27.1)	.028
Initial laboratory results, median (IQR)			
White blood cell count (× 10^3^/μL)	4.7 (1.9–20.2)	4.7 (1.9–16.0)	.95
Hemoglobin (g/dL)	9.0 (8.3–9.9)	9.7 (9.2–10.5)	<.001
Platelet (× 10^3^/μL)	52 (37–77)	60 (39–81)	.35
Absolute neutrophil count (/μL)	727 (206–2048)	594 (213-1691)	.60
Albumin (g/dL)	3.3 (3.0–3.8)	3.4 (3.0–3.8)	.52
Aspartate aminotransferase (IU/L)	22 (15–34)	21 (16–28.5)	.86
Alanine aminotransferase (IU/L)	18 (11–33)	18 (12–29)	.80
Alkaline phosphatase (IU/L)	66 (55–90.3)	76 (56–102)	.25
Total bilirubin (mg/dL)	0.6 (0.4–0.8)	0.5 (0.4–0.8)	.53
C-reactive protein (mg/dL)	1.48 (0.45–4.00)	1.23 (0.25–3.16)	.21
Lactate dehydrogenase (IU/L)	348 (221–629)	360 (223–618)	.87
Hematopoietic stem cell transplantation recipients	21 (13.0)	10 (11.8)	.79
Duration of neutropenia, median d (IQR)	26 (19–39)	28 (22–39)	.33
Duration of prophylaxis, median d (IQR)	23 (16–30)	Not applicable	

### 3.2 Invasive fungal disease

During chemotherapy, proven or probable invasive fungal infections occurred during treatment in 4 of the 162 patients (2.5%) from the posaconazole group and in 8 of the 85 patients (9.4%) from the no antifungal prophylaxis group. The incidence of proven or probable IFDs decreased significantly in the posaconazole prophylaxis group compared with the no prophylaxis group (2.5% [4/162] vs 9.4% [8/85], *P* = .03) (Table 2). However, there was no significant difference in possible IFD cases between the prophylaxis and no prophylaxis groups (4.3% [7/162] vs 3.5% [3/85], *P* > .99) (Table 2). In the posaconazole prophylaxis group, 3 patients had mold infection (2 *Aspergillus* and 1 mucormycosis) and 1 patient had invasive candidiasis. In the no prophylaxis group, 7 patients had mold infection (6 *Aspergillus* and 1 mucormycosis) and 1 patient had invasive candidiasis. The frequency of invasive mold infection was significantly higher in the no prophylaxis group than in the posaconazole prophylaxis group (8.2% vs 1.9%, *P* = .04), and *Aspergillus* was the most common pathogen in both groups. The lung was the most frequent site of IFD in the prophylaxis and no prophylaxis groups (1.9% [3/162] and 7.1% [6/85]).

**Table 2:** Incidence of invasive fungal disease between patients with posaconazole prophylaxis and those without prophylaxis.

	Posaconazole prophylaxis (n = 162)	No prophylaxis (n = 85)	*P* value
Patients with proven/probable IFD	4 (2.5)	8 (9.4)	.026
Mold	3 (1.9)	7 (8.2)	.035
Invasive aspergillosis	2 (1.2)	6 (7.1)	.021
Mucormycosis	1 (0.6)	1 (1.2)	>.99
Yeast	1 (0.6)	1 (1.2)	>.99
Invasive candidiasis	1 (0.6)	1 (1.2)	
*Candida glabrata*	1	0	
*Candida tropicalis*	0	1	
Patients with possible IFD	7 (4.3)	3 (3.5)	>.99
Site of infection			
Lung	3 (1.9)	6 (7.1)	
Sinus	0	1 (1.2)	
Blood	1 (0.6)	1 (1.2)	

### 3.3 Clinical outcomes

A total of 68 patients in the posaconazole prophylaxis group discontinued posaconazole owing to IFDs, empirical antifungal agent therapy, and adverse events. The frequency of therapeutic antifungal agent therapy owing to IFDs was higher in the no prophylaxis group than in the posaconazole prophylaxis group (9.4% vs 2.5%, *P* = .03) (Table 3). Empirical antifungal agent therapy was performed in 62 (38.3%) patients from the prophylaxis group and 55 (64.7%) patients from the no prophylaxis group (*P* < .01). Amphotericin B was the empirical antifungal agent most frequently administered to patients in the prophylaxis and no prophylaxis groups (69.4% [43/62] and 100% [55/55]). Events that occurred during the study period are described in Table 3. There were 2 cases (1.2%) of adverse events (elevated hepatic enzyme), resulting in the discontinuation of posaconazole. The duration from initial chemotherapy to therapeutic or empirical antifungal agent therapy was longer in the prophylaxis group than in the no prophylaxis group (18 days vs 11 days, *P* < .01). IFD-related mortality was similar between the 2 groups. One patient in the posaconazole prophylaxis group died owing to invasive pulmonary aspergillosis.

**Table 3:** Clinical outcome between patients with posaconazole prophylaxis and those without prophylaxis.

Clinical outcome	Posaconazole prophylaxis (n = 162)	No prophylaxis (n = 85)	*P* value
Use of targeted antifungal agents	4 (2.5)	8 (9.4)	.026
Use of empirical antifungal agents^∗^	62 (38.3)	55 (64.7)	<.001
Events during the study period			
Neutropenic fever	130 (80.2)	75 (88.2)	.11
Increases in C-reactive protein	159 (98.1)	85 (100.0)	.55
Increases in hepatic enzymes^†^	56 (34.6)	15 (17.6)	.005
Increases in total bilirubin	66 (40.7)	34 (40.0)	.91
Chest computed tomography	31 (19.1)	10 (11.8)	.14
Discontinuation of posaconazole due to adverse events associated with Posaconazole^‡^	2 (1.2)	Not applicable	
Duration from chemotherapy to antifungal therapy, median days (IQR)^§^	18 (13–22)	11 (7–16)	<.001
IFD-related mortality	1 (0.6)	0	> .99

## 4 Discussion

Patients with AML who undergo cytotoxic chemotherapy have a high incidence of IFDs. Many studies have shown that posaconazole prevents IFDs more effectively than other antifungal prophylaxis agents during chemotherapy and the neutropenic period in hematologic malignancy patients. Breakthrough IFDs during the exposure period of posaconazole prophylaxis was reported in 2% to 5% of AML patients during chemotherapy.^[8,10,15,16]^ At our center, the attending hematologists decide on the prophylactic strategy (i.e., posaconazole or none) depending on the presence of gastrointestinal symptoms or abnormal liver function. We thus had a unique chance to evaluate the incidence of breakthrough IFDs, the most common pathogen of IFDs during posaconazole prophylaxis, and the efficacy of posaconazole prophylaxis compared with no antifungal prophylaxis. As expected, posaconazole prophylaxis was associated with a significant reduction of IFDs in real practice. In addition, empirical antifungal treatment and therapeutic antifungal agent use were decreased after posaconazole prophylaxis. These findings indicate the importance of posaconazole prophylaxis because IFDs that occur during chemotherapy or the period of neutropenia can exhibit a fatal clinical course, lead to increased medical cost, and cause a delay in the chemotherapy schedule.

When breakthrough IFDs occur during posaconazole prophylaxis, several treatment options for empirical antifungal treatment have been suggested, which should be considered in relation to the potential of pathogens.^[17]^ In our study, the most common pathogen of breakthrough IFDs during prophylaxis was *Aspergillus* spp. In this context, a switch to mold-active antifungal agents such as voriconazole, isavuconazole, or liposomal amphotericin B for empirical antifungal treatment is reasonable in patients with posaconazole prophylaxis and suspected breakthrough infection.^[17]^ The most frequent site of IFD was the lungs (Table 2), probably because the most frequent pathogen was *Aspergillus* spp. and inhalation of spores plays a primary role in colonization.

Mucormycosis is a very aggressive life-threatening invasive fungal infection in immunocompromised patients and causes high morbidity and mortality in these patients.^[18,19]^ Posaconazole has limited activity against some of the mucorales species, and few cases of breakthrough mucormycosis infection have been reported in hematologic malignancy patients during posaconazole prophylaxis.^[20,21]^ However, the early detection of mucormycosis is difficult because there is no serologic marker or antigen detection marker. Imaging findings, such as the reversed halo sign, may be indicative but cannot provide proof of mucormycosis, so diagnosis should be verified by direct microscopy approaches such as culture and biopsies.^[22]^ There were 2 patients with mucormycosis infection in our study. One patient in the prophylaxis group was diagnosed through VATS biopsy, and another patient in the no prophylaxis group was diagnosed through endoscopic sinus surgery biopsy. Therefore, if IFD occurs during posaconazole prophylaxis, mucormycosis infection should be considered and early diagnosis should be performed. Taken together, given that there is non-negligible breakthrough mucormycosis in patients with posaconazole prophylaxis, the empirical antifungal use of liposomal amphotericin B or isavuconazole in those patients may be a cautious approach for broad coverage over aspergillosis and mucormycosis.

Our study has 3 limitations. First, there were several possible IFD cases suggesting invasive fungal infection on CT scan, but because of the patient's general condition and the decision of the hematologist, in many cases, bronchoalveolar lavage or VATS biopsy could not be performed. Therefore, it is possible that the incidence of IFD may be higher. Second, our study was a retrospective, nonrandomized clinical study. There were unmeasured confounders in the selection of posaconazole prophylaxis or no prophylaxis besides the attending hematologists’ preference. Third, posaconazole levels were not routinely measured to assess the bioavailability of posaconazole. Posaconazole oral suspension bioavailability is affected by food, mucositis, and gastric pH.^[23–25]^ However, delayed-release tablet formulation improves bioavailability, the serum concentration is higher with delayed-release tablet formulation than with suspension formulation, and acid suppression does not significantly affect absorption in tablet formulation.^[26–28]^ In this study, we used delayed-release tablet formulation, and a steady serum concentration of posaconazole can be expected.

In conclusion, our retrospective study on 247 patients with AML showed that patients who received posaconazole prophylaxis had a significantly lower incidence of breakthrough IFDs and the use of empirical or therapeutic antifungal agents compared with those who did not receive any antifungal prophylaxis. Invasive aspergillosis was the most common breakthrough IFD that occurred during posaconazole prophylaxis.

## Author contributions

**Supervision:** Eun-Ji Choi, Han-Seung Park, Sang-Oh Lee, Sang-Ho Choi, Yang Soo Kim, Jung-Hee Lee, Je-Hwan Lee, Kyoo-Hyung Lee, Sung-Han Kim.

**Writing – original draft:** Eunmi Yang.

**Writing – review & editing:** Eunmi Yang, Sung-Han Kim.
